# Association between triglyceride glucose index and odds of hip fracture in patients with or without type 2 diabetes: a cross-sectional study

**DOI:** 10.3389/fendo.2026.1879554

**Published:** 2026-07-13

**Authors:** Dan Zhao, Huiling Bai, Cuiping Zhao, Juzhong Tian, Yawen Bo

**Affiliations:** 1 Department of Endocrinology, The Second People’s Hospital of Changzhou, the Third Affiliated Hospital of Nanjing Medical University, Changzhou, Jiangsu, China; 2 Department of Geriatrics, The Second People’s Hospital of Changzhou, the Third Affiliated Hospital of Nanjing Medical University, Changzhou, Jiangsu, China; 3 Department of Stomatology, The Second People’s Hospital of Changzhou, the Third Affiliated Hospital of Nanjing Medical University, Changzhou, Jiangsu, China

**Keywords:** bone mineral density, cross-sectional study, hip fracture, triglyceride glucose index, type 2 diabetes

## Abstract

**Background:**

The triglyceride-glucose (TyG) index, an indicator of insulin resistance, has demonstrated variable correlations with fracture risk. This study sought to examine the correlation between TyG and the odds of hip fractures in Chinese individuals, both with and without type 2 diabetes (T2D).

**Methods:**

This retrospective cross-sectional study was performed at Changzhou Second People’s Hospital and involved 935 Chinese individuals, including 248 patients with hip fractures. TyG was calculated based on fasting glucose and triglyceride levels. Multivariate logistic regression models were used to investigate the association between TyG and the odds of hip fracture.

**Results:**

The odds of hip fractures was identical in both T2D and non-T2D groups (27.5% vs 26.3%, P = 0.734). After adjusting for all confounders, increasing TyG significantly reduced the odds of hip fracture in the group without T2D (odds ratio [OR] = 0.45; 95% confidence interval [CI]:0.25–0.83), but not in the T2D group (OR = 1.90; 95% CI:0.81–4.46). A significant multiplicative interaction was observed between T2D and TyG on the odds of hip fracture (p for interaction < 0.01). In addition, there is no association between TyG and bone mineral density (BMD) in either the T2D or without T2D participants (p > 0.05).

**Conclusion:**

Significant elevations in TyG were independently associated with a reduced odds of hip fractures in Chinese individuals without T2D, but not in those with T2D. These findings underscore the necessity of considering diabetes status when evaluating the TyG index as a metabolic determinant of hip fracture odds in clinical practice.

## 1 Introduction

Hp fractures pose a major public health issue related with great morbidity and death (1). The global odds of hip fractures is projected to increase from 1.26 million in 1990 to 4.5 million by 2050 (2). The one-year mortality rate following a hip fracture surpasses 20%-30% (3, 4) and is projected to rise by 2% annually (3). The typical expense for treating a hip fracture in China has reached ¥53,440, about equivalent to the complete annual revenue of several Chinese households (5). With the ongoing rise and aging of the population, the health burden of hip fractures is anticipated to escalate, presenting a significant socioeconomic and healthcare concern globally, particularly in China.

Insulin resistance (IR) is a metabolic disorder marked by diminished sensitivity and responsiveness to insulin, leading to compromised glucose absorption and utilization by peripheral tissues, including skeletal muscle and adipose tissue (6). To enable a more straightforward and convenient evaluation of IR, Luis E. et al. introduced the triglyceride-glucose (TyG) index, derived from fasting glucose and triglyceride concentrations (7). Numerous studies have demonstrated that the TyG index is similarly precise to the hyperinsulinemic-euglycemic clamp (HECT) and the homeostasis model assessment of insulin resistance (HOMA-IR) in evaluating IR (8–10).

Recent studies, however, revealed incongruous findings about the correlation between the TyG index and bone fractures (11). A study from the National Health and Nutrition Examination Survey (NHANES) reveals that heightened TyG scores in U.S. people aged 20 and above correlate with a greater risk of spinal fractures (12). A cohort research in China indicated that a higher TyG index correlates with a reduced incidence of vertebral fractures in the general population and among individuals without diabetes (13). The Kailuan cohort posits that a high cumTyG index constitutes a risk factor for fragility fractures (14). The associations of TyG and hip fracture remain less explored, especially in patients with or without type 2 diabetes.

This study sought to examine the strong relationship between TyG index and the odds of hip fractures in Chinese individuals, while accounting for all confounding variables. An additional study was performed to examine the association between T2D and non-T2D strata.

## 2 Methods

### 2.1 Study population

**Figure 1:**
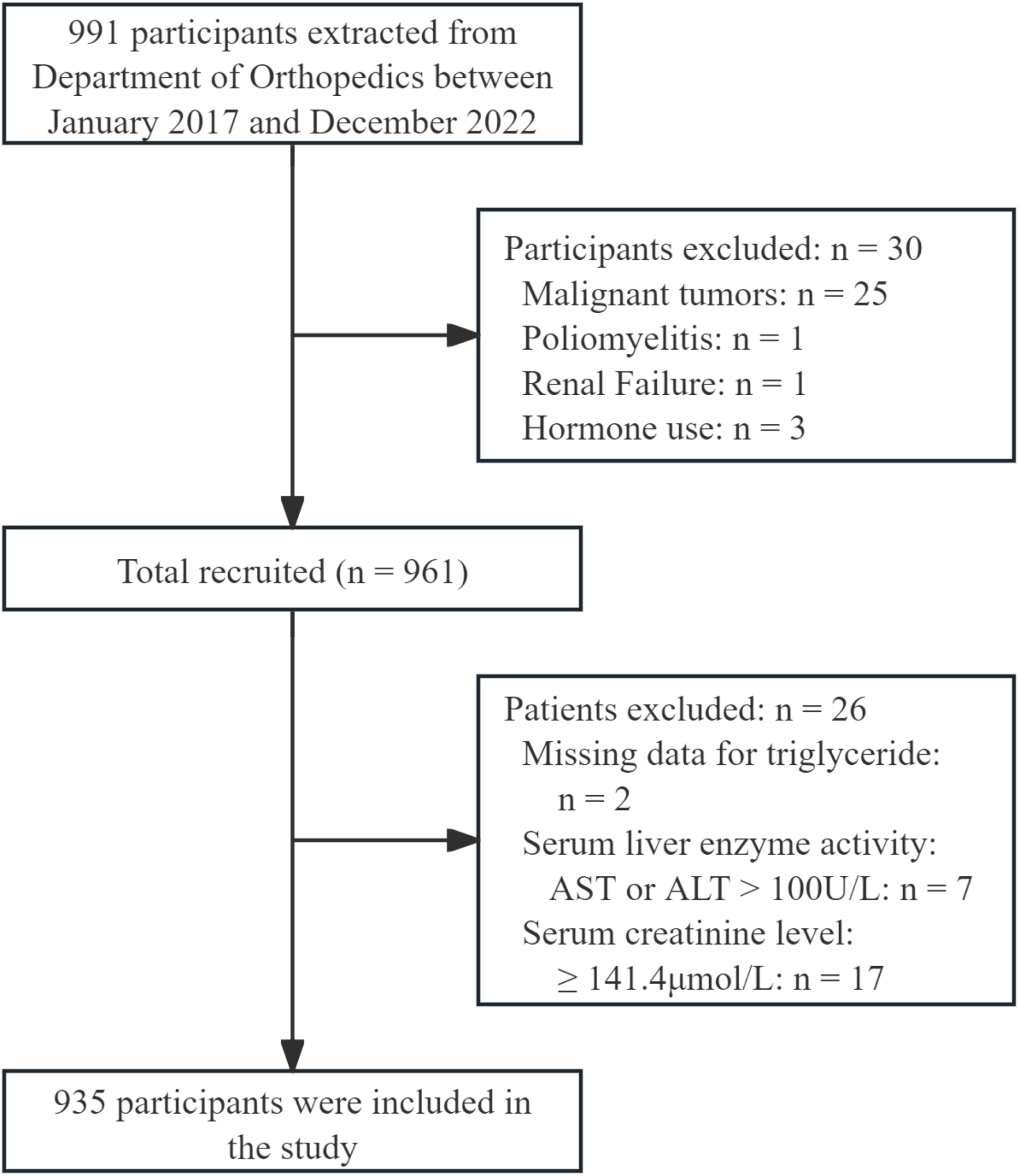
Flowchart depicting the screening and enrolling procedures for study participants. AST, aspartate aminotransferase; ALT, alanine aminotransferase.

This retrospective cross-sectional study was performed in the Department of Orthopedics at the Second People’s Hospital of Changzhou, the Third Affiliated Hospital of Nanjing Medical University, Jiangsu, China. The source population comprised all adult patients (aged ≥ 18 years) who were consecutively admitted to the orthopedic inpatient service between January 2017 and December 2022. Participants were selected using consecutive sampling with no additional selection procedures applied. The criteria for participant acceptance and exclusion were previously outlined (15). Individuals with malignant neoplasms, poliomyelitis, renal insufficiency, hormone administration, absent triglyceride data, raised blood liver enzyme activity, or heightened serum creatinine levels (n = 56) were removed. Hip fractures were verified via a physician’s assessment of the radiology reports, involving a review of 935 participants, of whom 248 had hip fractures (**Figure 1**).

### 2.2 Assessment of clinical and laboratory metrics

Data regarding age, weight, height, and diabetes history was gathered from all participants in the study (16). Weight (kg) and height (m) were measured with a scale while participants wore light clothing and no shoes (RGZ-120-RT; Hengqi Inc., China). The body mass index (BMI; kg/m²) was computed by dividing weight (kg) by the square of height (m²). Diabetes is characterized by a fasting glucose level exceeding 7.0 mmol/L, a glycosylated hemoglobin A1c (HbA1c) greater than 6.5% (17), or a self-reported diagnosis of diabetes by a physician.

Following an overnight fast of at least 8 hours, blood samples were collected from all individuals within 24 hours of admission. Blood counts were evaluated with a self-service hematological analyzer (XN-2800, Sysmex Inc.). These consist of white blood cells (WBC), red blood cells (RBC), and platelets (PLT). Furthermore, the Siemens ADVIA-2400 was employed to evaluate the following parameters: ALT, AST, alkaline phosphatase (ALP), albumin (ALB), blood urea nitrogen (BUN), creatinine (CCR), triglycerides (TG), high-density lipoprotein cholesterol (HDL-C), low-density lipoprotein cholesterol (LDL-C), fasting plasma glucose (FPG), and C-reactive protein (CRP). HbA1c levels were evaluated using TOSOH G8-90SL. The erythrocyte sedimentation rate (ESR) was measured using an ALIFAX TEST1–2730 instrument.

TyG (Equation 1) can be defined as follow (7):

TyG=ln[triglyceride(mg/dL)xfasting plasma glucose(mg/dL)/2] (1).

### 2.3 Bone mineral density measurements

DXA scans of the hips were conducted utilizing the Hologic Discovery Wi (Hologic Inc., Bedford, MA, USA). All scanners were operated by licensed personnel. The short-term coefficient of variation (CV) for femoral neck BMD measurements in our center was <1.0%, which is within the manufacturer’s specified precision limits (femoral neck: 1.4%) (18). The examined bone density was characterized as the predicted (areal) BMD of the left femoral neck in subjects devoid of fractures. Patients who had suffered a hip fracture had their contralateral femoral necks measured. Bone density measurements were performed on all patients within 24 hours of admission.

### 2.4 Statistical analysis

Patients were classified into two groups depending on T2D incidence: mean ± standard deviation (SD) for normally distributed data, and median with interquartile range for skewed variables. Proportions (%) represented categorical variables. Chi-square (categorical variables), independent sample t-test (normal distribution), and Kruskal–Wallis (skewed distribution) tests compared variables between groups. The mean or median values were used to replace missing values for continuous variables with missing values less than 2%.

Non-adjusted and multivariate-adjusted models were applied. Expert opinion, prior research, and all significant factors reported in the univariate analysis (p <0.05) were taken into consideration while choosing these confounders. To investigate the association between TyG and the odds of hip fracture, we first fitted multivariate logistic regression models in the overall study population. The results were reported as odds ratios (ORs) and 95% CIs. Three models were developed: Model I was adjusted for age, BMI, and gender; model II was adjusted for model I + WBCs, RBCs, PLTs, ALT, ALB, LDL-C, CRP, and ESR; model III was adjusted for model II + BMD. Analyses of interaction and stratification were conducted according to gender, the existence of T2D, age, and BMI. Stratified multivariate regression analyses were conducted in participants with and without T2D, respectively.

Restricted cubic spline (RCS) regression was performed to visualize the overall dose-response relationship and T2D status-stratified curves between TyG and the odds of hip fracture, with 4 knots placed at the 5th, 35th, 65th, and 95th percentiles.

After dichotomizing TyG into low and high groups based on median value, we performed multivariate logistic regression analysis to assess the joint effects of TyG with gender and T2D on the odds of hip fracture.

Additionally, we used multivariate linear regression models to examine association of TyG with BMD. The results were reported as βs and 95% CIs.

Analysis was conducted utilizing R 4.2.2 (https://www.r-project.org/; The R Foundation, Vienna, Austria) and Free Statistics software (version 2.1.1; Beijing FreeClinical Medical Technology Co., Ltd, Beijing, China). Statistical significance was defined as a two-sided p-value of < 0.05.

## 3 Results

### 3.1 Baseline study participant characteristics

A total of 935 participants (293 men and 642 women) were enrolled. Baseline clinical and biochemical characteristics of participants were stratified by incident T2D are presented in **Table 1**. The mean age of the participants was 68.3 years old (standard deviation [SD] 10.5), ranging from 31 to 99 years. The mean BMI value was 25.1 kg/m^2^ (SD 3.9). Compared with the participants without T2D, participants with T2D were older, they exhibited higher levels of TG, FPG, HbA1c, and TyG. Notably, despite these metabolic differences, BMD and the odds of hip fracture were comparable between the two groups. The odds of hip fractures was identical in both T2D and non-T2D groups (27.5% vs 26.3%, P = 0.734).

**Table 1:** Baseline characteristics of participants.

Variables	Total	Without T2D	With T2D	P-value
	(n = 935)	(n = 731)	(n = 204)	
gender, %				0.855
Male	293 (31.3)	228 (31.2)	65 (31.9)	
Female	642 (68.7)	503 (68.8)	139 (68.1)	
Age, years	68.3 ± 10.5	67.6 ± 10.8	70.7 ± 8.9	< 0.001
Weight, kg	62.9 ± 11.1	62.5 ± 10.8	64.4 ± 12.1	0.030
Height, m	1.6 ± 0.1	1.6 ± 0.1	1.6 ± 0.1	0.868
BMI, kg/m^2^	25.1 ± 3.9	25.0 ± 3.8	25.7 ± 4.2	0.027
WBC, 10^9^/L	6.8 ± 3.5	6.5 ± 2.2	7.7 ± 6.1	< 0.001
RBC, 10^9^/L	4.3 ± 0.5	4.3 ± 0.5	4.4 ± 0.5	0.187
PLT, 10^9^/L	211.9 ± 64.9	210.4 ± 65.6	217.0 ± 62.3	0.204
ALT, U/L	19.4 ± 11.8	19.0 ± 11.6	20.6 ± 12.5	0.093
AST, U/L	21.0 ± 8.6	21.2 ± 8.5	20.2 ± 8.8	0.140
ALP, U/L	80.6 ± 26.6	79.9 ± 26.7	82.9 ± 26.5	0.162
ALB, g/L	42.5 ± 4.3	42.5 ± 4.4	42.8 ± 3.9	0.359
BUN, mmol/L	6.1 ± 3.2	6.0 ± 3.5	6.5 ± 2.1	0.059
CCR, μmol/L	62.7 ± 16.9	61.8 ± 16.0	65.8 ± 19.2	0.003
TG, mmol/L	1.6 ± 0.9	1.5 ± 0.8	1.8 ± 1.2	< 0.001
HDL-C, mmol/L	1.4 ± 0.3	1.4 ± 0.3	1.3 ± 0.3	0.001
LDL-C, mmol/L	2.6 ± 0.8	2.7 ± 0.8	2.5 ± 0.8	0.044
FPG, mmol/L	6.0 ± 1.8	5.4 ± 0.8	8.2 ± 2.6	< 0.001
CRP, mg/L	5.0 (3.5, 14.5)	5.0 (3.4, 14.2)	5.2 (3.9, 17.8)	0.141
HbA1c, %	6.2 ± 1.1	5.8 ± 0.5	7.6 ± 1.4	< 0.001
ESR, mm/h	21.0 (11.0, 34.0)	20.0 (10.0, 31.5)	25.0 (13.0, 39.2)	< 0.001
BMD, g/cm^2^	0.8 ± 0.2	0.8 ± 0.2	0.8 ± 0.2	0.854
TyG	8.8 ± 0.6	8.6 ± 0.5	9.1 ± 0.6	< 0.001
Fracture, n (%)	248 (26.5)	192 (26.3)	56 (27.5)	0.734

data presented are mean ± SD, median (Q1–Q3), or N (%).

T2D, type 2 diabetes; BMI, body mass index; WBC, white blood cell; RBC, red blood cell; PLT, platelet; ALT, alanine aminotransferase; AST, aspartate aminotransferase; ALP, alkaline phosphatase; ALB, albumin; BUN, blood urea nitrogen; CCR, creatinine; TG, triglycerides; HDL-C, high-density lipoprotein cholesterol; LDL-C, low-density lipoprotein cholesterol; FPG, fasting plasma glucose; HbA1c, glycosylated hemoglobin type-A1c; CRP, C-reactive protein; ESR, erythrocyte sedimentation rate; BMD, bone mineral density; TyG, triglyceride glucose index

### 3.2 Associations between TyG and odds of hip fracture in patients with or without T2D

The odds of hip fracture decreased with higher TyG(OR = 0.42; 95% CI:0.31–0.55) in the univariate logistic regression analyses. Age, BMI, WBC, RBC, PLT, ALT, ALB, LDL-C, CRP levels, and ESR were associated with the odds of hip fracture, which was adjusted in model II (**Supplementary Table 1**).

**Table 2** presents the results of the multivariate logistic regression analysis. After adjusting for age, BMI, and gender, the association remained statistically significant. In model II, increase in TyG (per 1 dimensionless) was associated with a 34% decrease in the odds of hip fracture (OR = 0.66; 95% CI:0.44–0.99). However, upon further adjustment for BMD in model III, the association between TyG and fracture risk remained inverse but lost statistical significance (OR 0.66, 95% CI 0.43–1.01, P = 0.054). Consistent results were observed when TyG was analyzed as tertiles.

**Table 2:** Association between TyG and the odds of hip fracture.

Variable	Nonadjusted	P-value	model I	P-value	model II	P-value	model III	P-value
TyG (continuous)	0.42 (0.31~0.55)	<0.001	0.62 (0.45~0.84)	0.002	0.66 (0.44~0.99)	0.046	0.66 (0.43~1.01)	0.054
TyG (tertile)								
T1	Reference		Reference		Reference		Reference	
T2	0.36 (0.25~0.51)	<0.001	0.48 (0.32~0.71)	<0.001	0.54 (0.32~0.91)	0.021	0.59 (0.34~1.01)	0.055
T3	0.30 (0.21~0.43)	<0.001	0.53 (0.35~0.80)	0.003	0.65 (0.37~1.14)	0.136	0.67 (0.38~1.20)	0.177
Trend.test	0.53 (0.44~0.64)	<0.001	0.70 (0.57~0.87)	0.001	0.79 (0.59~1.05)	0.100	0.80 (0.60~1.08)	0.141

Notes: data are presented as ORs and 95% CIs.

Adjusted model I was adjusted for age, body mass index, and gender; adjusted model II was adjusted for model I + white blood cells, red blood cells, platelets, alanine aminotransferase, albumin, low-density lipoprotein cholesterol, C-reactive protein, and erythrocyte sedimentation rate; adjusted model III was adjusted for model II + bone mineral densityAbbreviations: TyG, triglyceride glucose index

**Figure 2:**
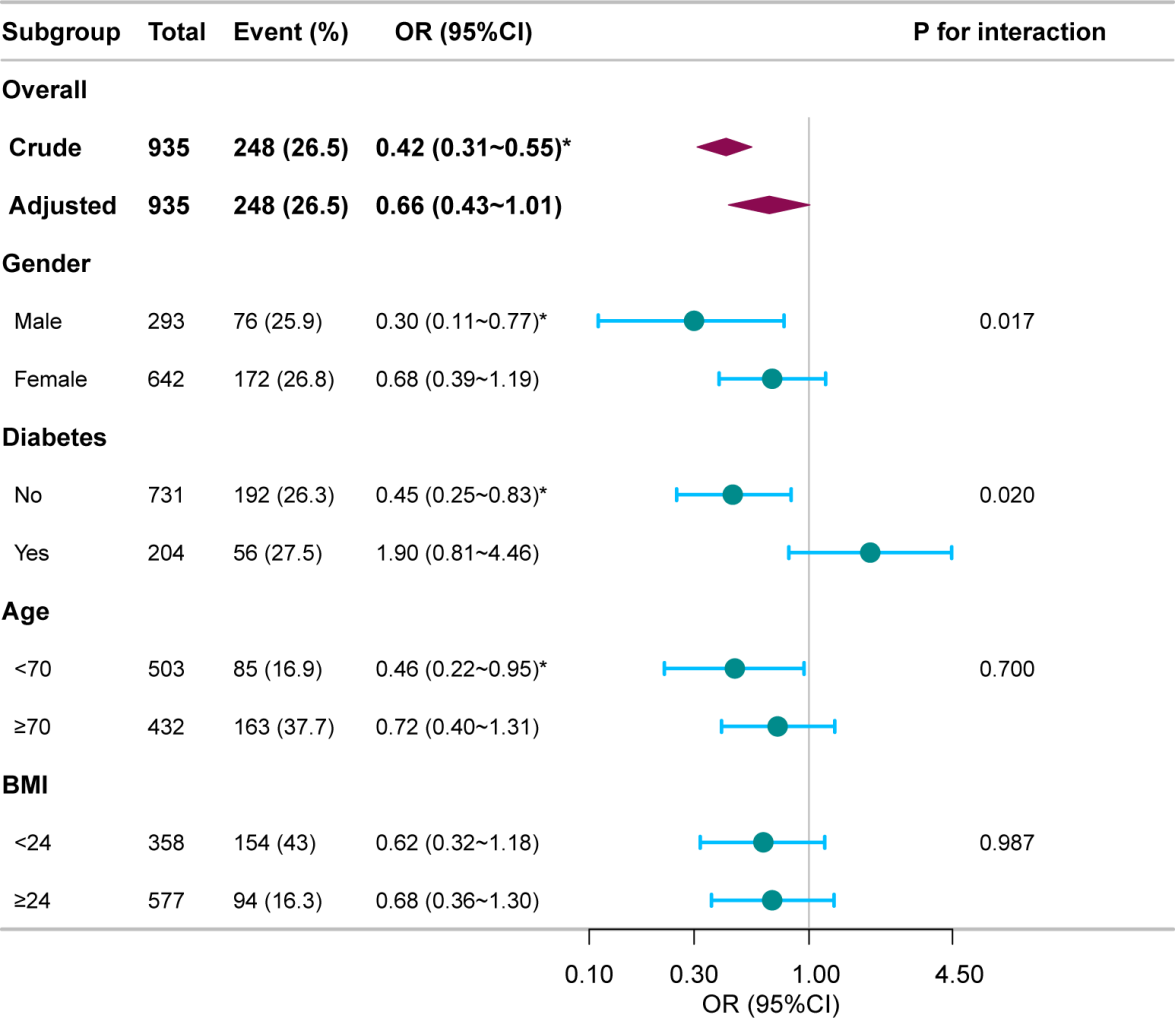
Association between TyG and the odds of hip fracture in Subgroup analyses based on gender, diabetes, age, and BMI. Each stratification adjusted for all factors (age, body mass index, gender, white blood cells, red blood cells, platelets, alanine aminotransferase, albumin, low-density lipoprotein cholesterol, C-reactive protein, erythrocyte sedimentation rate and bone mineral density) except the stratification factor itself. BMI, body mass index; TyG, triglyceride glucose index. ^*^p < 0.05.

Subgroup analyses were performed to further examine the influence of gender, diabetes, age, and BMI on study outcomes. The outcomes of these investigations are presented in **Figure 2**. Trends in effect sizes were consistent across gender, age, and BMI groups. Nevertheless, increase in TyG (per 1 dimensionless) was associated with a 90% higher odds of hip fracture in diabetes group (OR = 1.90; 95% CI:0.81–4.46), although the association was not statistically significant. Some interactions were found between gender and diabetes among participants (p for interaction < 0.05).

**Table 3** presents the association between TyG index and hip fracture odds stratified by T2D status. In participants without T2D, high TyG was consistently associated with reduced fracture risk across all models. Conversely, in the T2D group, TyG showed no significant association with fracture risk in any model. After adjusting for all confounders, increasing TyG significantly reduced the odds of hip fracture in the group without T2D (OR = 0.45; 95% CI:0.25–0.83), but not in the T2D group (OR = 1.90; 95% CI:0.81–4.46).

**Table 3:** Association between TyG and the odds of hip fracture in patients with or without T2D.

Model	Without T2D (n = 731)		With T2D (n = 204)	
	OR (95% CI)	P-value	OR (95% CI)	P-value
Nonadjusted	0.20 (0.14~0.30)	<0.001	1.14 (0.69~1.89)	0.609
model I	0.34 (0.22~0.53)	<0.001	1.28 (0.74~2.23)	0.379
model II	0.43 (0.24~0.78)	0.005	2.12 (0.94~4.79)	0.072
model III	0.45 (0.25~0.83)	0.011	1.90 (0.81~4.46)	0.140

Adjusted model I was adjusted for age, body mass index, and gender; adjusted model II was adjusted for model I + white blood cells, red blood cells, platelets, alanine aminotransferase, albumin, low-density lipoprotein cholesterol, C-reactive protein, and erythrocyte sedimentation rate; adjusted model III was adjusted for model II + bone mineral density. Abbreviations: TyG, triglyceride glucose index; T2D, type 2 diabetes.

### 3.3 Restricted cubic spline curves

**Figure 3:**
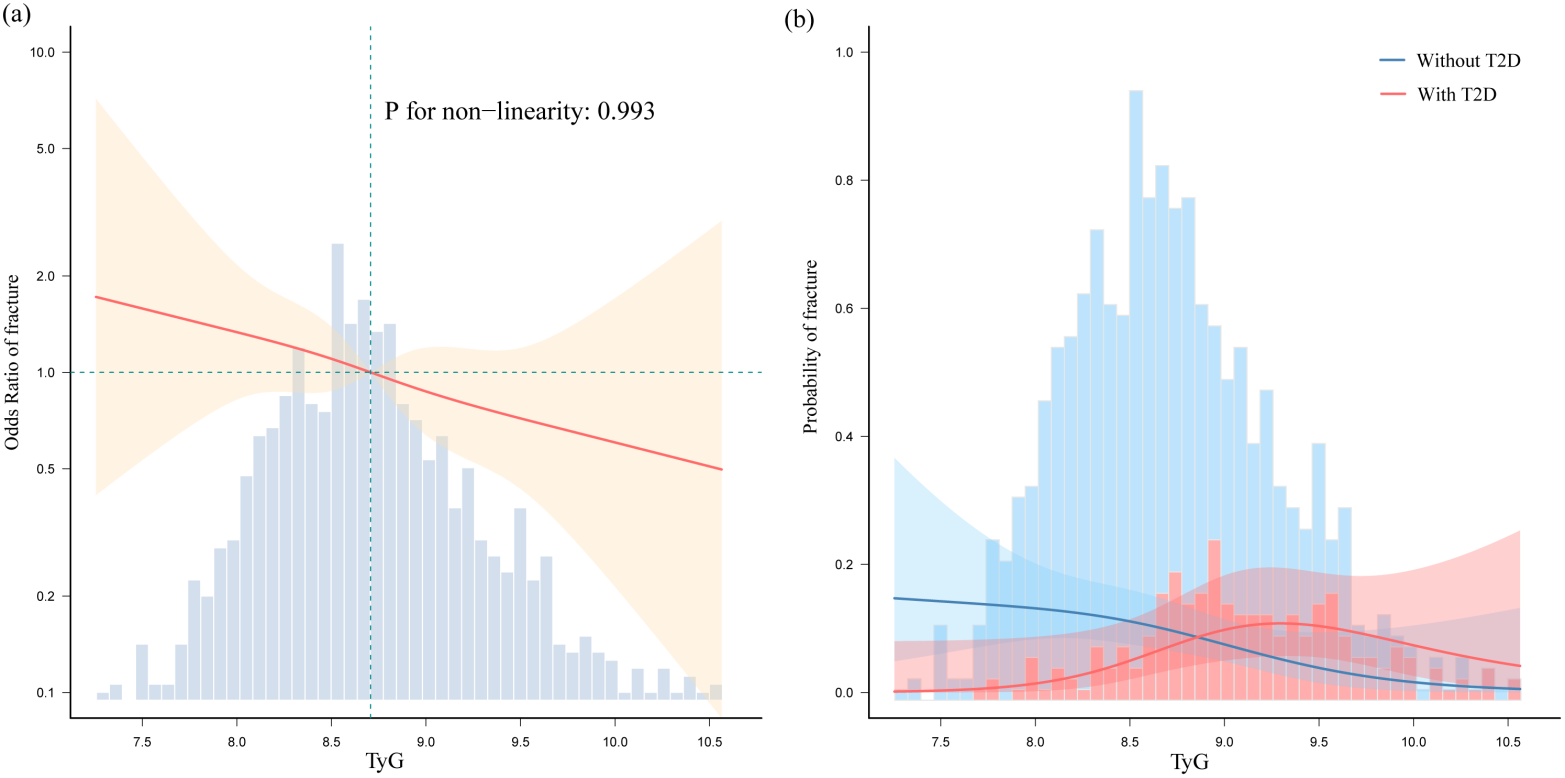
**(a)** A linear association between TyG and the odds of hip fracture. **(b)** T2D status-stratified restricted cubic spline curves for the association between TyG and fracture probability. Adjustment factors included age, body mass index, gender, white blood cells, red blood cells, platelets, alanine aminotransferase, albumin, low-density lipoprotein cholesterol, C-reactive protein, and erythrocyte sedimentation rate and bone mineral density. Abbreviations: T2D, type 2 diabetes; TyG, triglyceride glucose index.

**Figure 3a** demonstrates a significant linear inverse association between TyG index and hip fracture odds (p for nonlinearity > 0.05). Stratified analyses by T2D status revealed divergent patterns in **Figure 3b**. Fracture probability decreased consistently with increasing TyG in the group without T2D, whereas no apparent association was observed in those with T2D, corroborating the significant interaction identified in the main analysis.

### 3.4 Multiplicative interaction between T2D and TyG on the odds of hip fracture

**Figure 4:**
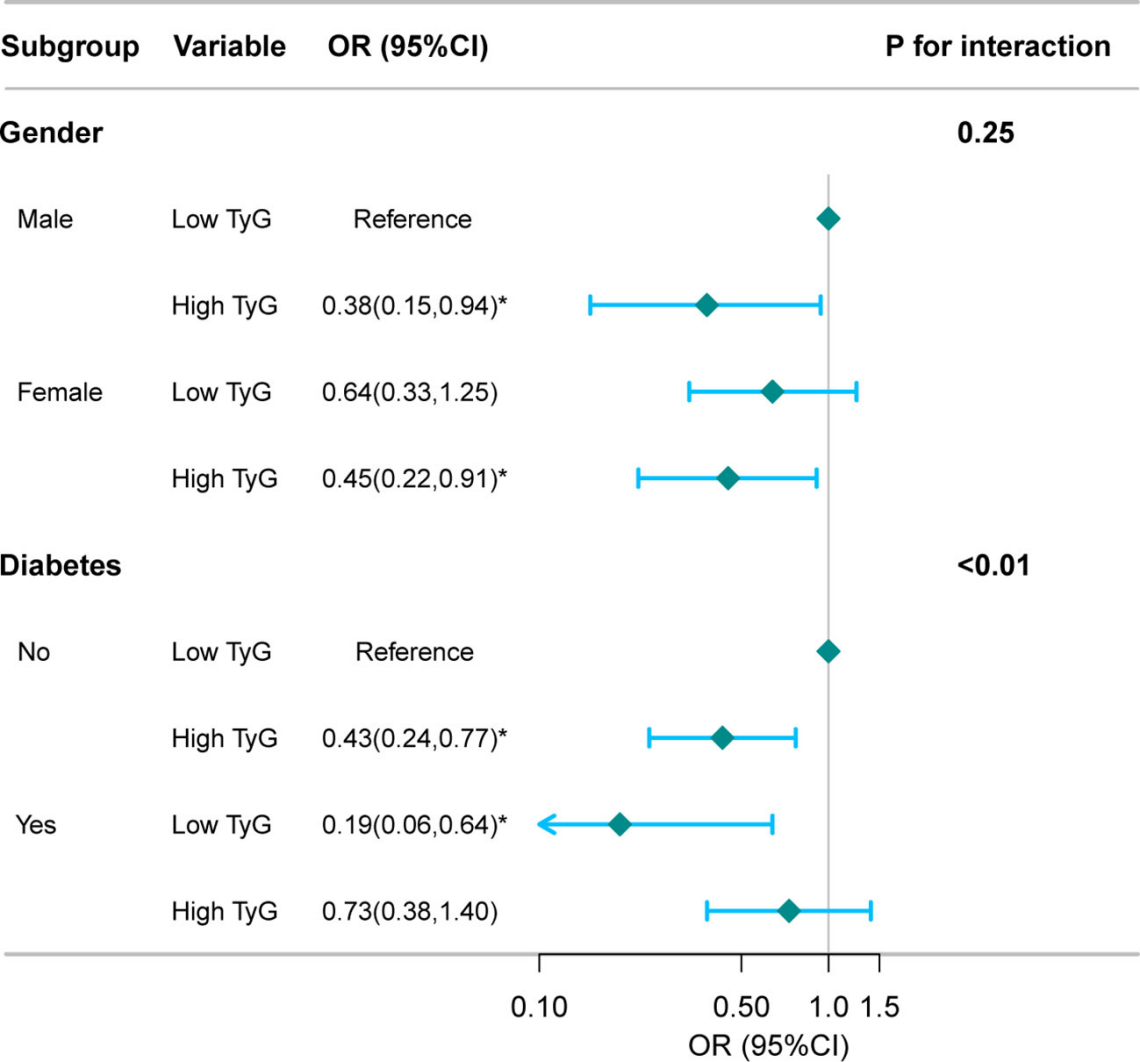
Joint associations (OR, 95% CI) of TyG (low or high) and gender(male or female), diabetes status (no or yes) with the odds of hip fracture. Adjustment factors included age, body mass index, gender, white blood cells, red blood cells, platelets, alanine aminotransferase, albumin, low-density lipoprotein cholesterol, C-reactive protein, and erythrocyte sedimentation rate and bone mineral density. TyG, triglyceride glucose index. ^*^p < 0.05.

A significant multiplicative interaction was observed between T2D and TyG on the odds of hip fracture in **Figure 4**. No such interaction was observed for gender (P = 0.25).

### 3.5 Secondary analysis

In multivariate linear regression analysis, TyG was positively associated with femoral neck BMD(β = 0.03; 95% CI:0.01–0.05). However, the association was not statistically significant in both models I and II (p > 0.05). This association was not observed in either the T2D or without T2D participants (**Supplementary Table 2**).

## 4 Discussion

This retrospective cross-sectional study comprised 935 Chinese adults, of whom 248 sustained hip fractures. Our investigation reveals a strong negative correlation between the TyG and hip fracture odds in Chinese adults without T2D but not among those with T2D. Significant multiplicative interaction was found between TyG and diabetes status on risks of hip fracture. In addition, there is no association between TyG and BMD.

To date, evidence regarding the association of TyG with fractures risks remains controversial. Several studies have reported divergent findings regarding this association in the general population. In a cross-sectional NHANES analysis, Geng et al. found that American adults with a higher TyG index had a higher risk of spine fracture (OR: 1.63, 95% CI: 1.15-2.30) (12). Conversely, Wei et al. performed a longitudinal cohort study in older Chinese adults and found that a greater TyG index was associated with a lower risk of vertebral fracture (HR 0.56, 95% CI 0.35–0.90) (13). Recently, Yao et al. found that a high cumulative exposure to TyG index (cumTyG) was an independent risk factor for fragility fracture in a large prospective cohort of 55,824 participants with a mean follow-up of 11.35 years (HR 1.30, 95% CI 1.04–1.61) (14). These inconsistent findings may be attributable to several factors, including differences in study design, fracture sites examined, ethnic backgrounds, and the metrics used for assessing insulin resistance. Importantly, none of these three studies incorporated BMD into their adjusted models.

In our study, increase in TyG was associated with a 34% decrease in the odds of hip fracture (OR 0.66, 95% CI 0.44–0.99) after adjustment for demographic and clinical covariates. This observation aligns directionally with the protective effect described by Wei et al (13). Nevertheless, after additional adjustment for BMD in the fully adjusted model, this inverse correlation remained in direction but ceased to be statistically significant (OR 0.66, 95% CI 0.43–1.01, P = 0.054). This pattern suggests that BMD may partially mediate or confound the relationship between TyG and hip fracture. The lack of a direct association between TyG and BMD in our cohort suggests that the observed attenuation may reflect residual confounding rather than a true mediating effect.

The presence of a significant multiplicative interaction formally confirms that the effect of TyG on hip fracture odds is modified by diabetes status, rather than representing a chance subgroup finding. After adjusting for all confounding factors, including BMD, a higher TyG index was consistently associated with reduced fracture risk. Wei et al. observed a comparable protective association in persons devoid of diabetes (aHR 0.44, 95% CI 0.21–0.95) (13). Pan et al. indicated that TyG was positively correlated with the risk of fragility fractures (HR 1.636, 95% CI 1.042–2.570) in 220 postmenopausal older women with T2DM and osteoporosis (19). However, the corresponding estimate in our T2D subgroup, while showing a similar trend (OR 1.90), was imprecise and did not reach statistical significance (95% CI 0.81–4.46), likely reflecting the limited sample size (n = 204). Consequently, this result should be considered exploratory and requires validation in larger diabetic cohorts.

To explore the biological plausibility of our findings, we synthesized established mechanistic evidence from prior studies to formulate a hypothesis regarding the differential effects of TyG on bone metabolism across glycemic states. In individuals without T2D, a higher TyG index may serve as a surrogate for enhanced adiposity and nutritional reserve, which could confer biomechanical protection against hip fracture through greater soft tissue padding and higher body weight (20). Mild insulin resistance in the non-diabetic range may be associated with compensatory hyperinsulinemia, which exerts anabolic effects on bone. Insulin has been demonstrated to boost osteoblast proliferation and differentiation via the MAPK and PI3K signaling pathways, augment alkaline phosphatase activity, enhance type I collagen production, and promote osteocalcin expression and mineralized nodule formation in osteoblastic cells (21). Furthermore, insulin decreases osteoclast activity by reducing RANKL signaling and suppressing osteoclastogenesis (22). Conversely, in established T2D, the TyG index likely captures more severe and chronic metabolic perturbations that fundamentally alter the skeletal effects of insulin resistance. Advanced glycation end-products (AGEs) accumulate in diabetic bone, resulting in increased non-enzymatic crosslinking inside collagen fibrils. This process impedes intrafibrillar mineralization and causes disordered mineral deposition, ultimately diminishing bone strength despite normal or BMD (23).

Our study has several limitations. First, the retrospective cross-sectional design precludes the establishment of a causal relationship between the TyG index and hip fracture odds, and prospective studies are warranted to validate our findings. Second, the TyG index, as a surrogate marker of insulin resistance, was calculated from a single fasting measurement and may not capture dynamic metabolic fluctuations. Third, although we adjusted for major confounding factors, information on vitamin D status, physical activity, and history of falls was not available for inclusion in the analysis. Fourth, the relatively small sample size in the T2D subgroup (n = 204) resulted in a wide confidence interval, which may have limited the statistical power to detect a significant association between TyG and hip fracture in this stratum. Finally, this is a single-center retrospective analysis, and the original patient data were deficient in records pertaining to history of falls, physical activity, prior fractures, vitamin D levels, calcium intake, antidiabetic medications, lipid-lowering medications, and medications affecting bone metabolism. These cross-sectional findings require confirmation in prospective, multicenter cohorts before any clinical use.

## 5 Conclusions

Significant elevations in TyG were independently associated with a reduced odds of hip fractures in Chinese individuals without T2D, but not in those with T2D. These findings underscore the necessity of considering diabetes status when evaluating the TyG index as a metabolic determinant of hip fracture odds in clinical practice.

## Data Availability

The raw data supporting the conclusions of this article will be made available by the authors, without undue reservation.
