# Supplementary material for: Association between triglyceride glucose index and odds of hip fracture in patients with or without type 2 diabetes: a cross-sectional study
Source: Front Endocrinol (Lausanne). 2026 Jul 13;17:1879554. doi: 10.3389/fendo.2026.1879554 (PMC13402920; doi:10.3389/fendo.2026.1879554)
Supplement: Supplementary file 1 [file Table1.docx]

Supplementary Material

**Supplementary Table 1** Univariate analysis for the risk of hip fracture

| Variable | OR (95% CI) | P_value |
| --- | --- | --- |
| Age, years | 1.07 (1.05~1.08) | <0.001 |
| Weight, kg | 0.95 (0.93~0.96) | <0.001 |
| Height, per 0.1m | 1.65 (1.37~1.99) | <0.001 |
| BMI, kg/m^2^ | 0.77 (0.73~0.81) | <0.001 |
| WBC, 10^9^/L | 1.46 (1.35~1.57) | <0.001 |
| RBC, 10^9^/L | 0.30 (0.22~0.41) | <0.001 |
| PLT, per 10^^^10^9^/L | 0.95 (0.93~0.98) | <0.001 |
| ALT, U/L | 0.97 (0.96~0.99) | 0.001 |
| AST, per 10 U/L | 1.04 (0.88~1.22) | 0.683 |
| ALP, per 10 U/L | 1.05 (1.00~1.11) | 0.061 |
| ALB, g/L | 0.77 (0.74~0.81) | <0.001 |
| BUN, mmol/L | 1.00 (0.95~1.04) | 0.858 |
| CCR, μmol/L | 1.01 (1.00~1.02) | 0.055 |
| TG, mmol/L | 0.35 (0.26~0.46) | <0.001 |
| HDL-C, mmol/L | 0.99 (0.64~1.52) | 0.946 |
| LDL-C, mmol/L | 0.50 (0.41~0.62) | <0.001 |
| FPG, mmol/L | 1.17 (1.09~1.27) | <0.001 |
| CRP, mg/L | 1.07 (1.06~1.09) | <0.001 |
| HbA1c, % | 1.04 (0.92~1.19) | 0.508 |
| ESR, mm/h | 1.02 (1.02~1.03) | <0.001 |
| BMD, per 0.1 g/cm^2^ | 0.47 (0.42~0.54) | <0.001 |
| TyG | 0.42 (0.31~0.55) | <0.001 |

Note: data are presented as ORs and 95% CIs

Abbreviations:BMI, body mass index; WBC, white blood cell; RBC, red blood cell; PLT, platelet; ALT, alanine aminotransferase; AST, aspartate aminotransferase; ALP, alkaline phosphatase; ALB, albumin; BUN, blood urea nitrogen; CCR, creatinine; TG, triglycerides; HDL-C, high-density lipoprotein cholesterol; LDL-C, low-density lipoprotein cholesterol; FPG, fasting plasma glucose; HbA1c, glycosylated hemoglobin type-A1c; CRP, C-reactive protein; ESR, erythrocyte sedimentation rate; BMD, bone mineral density; TyG, triglyceride glucose index.

**Supplementary Table 2** Association between TyG and BMD

| Model | Total (n = 935) | | Without T2D (n = 731) | | With T2D (n = 204) | |
| --- | --- | --- | --- | --- | --- | --- |
|  | β (95% CI) | P_value | β (95% CI) | P_value | β(95% CI) | P_value |
| Nonadjusted | 0.03 (0.01~0.05) | 0.004 | 0.06 (0.03~0.08) | <0.001 | -0.03 (-0.07~0.01) | 0.129 |
| model I | 0.01 (-0.01~0.02) | 0.386 | 0.02 (0.00~0.04) | 0.042 | -0.02 (-0.06~0.01) | 0.204 |
| model II | 0.00(-0.01~0.02) | 0.703 | 0.01 (-0.01~0.03) | 0.185 | -0.03 (-0.07~0.01) | 0.112 |

Notes: data are presented as βs and 95% CIs

Adjusted model I was adjusted for age, body mass index, and gender; adjusted model II was adjusted for model I + white blood cells, red blood cells, platelets, alanine aminotransferase, albumin, low-density lipoprotein cholesterol, C-reactive protein, and erythrocyte sedimentation rate.

Abbreviations: TyG, triglyceride glucose index; BMD, bone mineral density; T2D, type 2 diabetes
